# The Pan-American Medical Congress

**Published:** 1900-12

**Authors:** 


### THE PAN-AMERICAN MEDICAL CONGRESS.

Dr. H. A. West, Galveston, Secretary Texas State Medical Association, has been appointed secretary of the section on Medical Jurisprudence to represent the United States at the approaching meeting of the Pan-American Medical Congress, which will be held in Havana, Cuba, December 26 to 29. See notice of meeting elsewhere in this issue. Those who intend contributing papers to the section on Medical Jurisprudence should promptly send the titles of their papers to Dr. West. We trust that Texas will be fully represented at this meeting. That she will be well represented in the person of our handsome secretary—well, that's settled.

Dr. West has also been elected a member of the Auxiliary Committee of the Pan-American Medical Congress. This is a judicious selection. Dr. West will have it in his power to facilitate the success of the congress, and we know that he will be pleased to co-operate with Texas physicians, especially, who may wish to attend the meeting, or to contribute paeprs to any of the sections. If the titles of any papers for any of the sections are sent to the doctor he will see that they reach the hands of the associate secretary without delay. There is a round trip rate to Havana, via New Orleans,—the Morgan Line,—for $50. This includes state room and meals on board, going and coming. Returning vessel will reach New Orleans January 3rd. Write Dr. West for further particulars.

---

THE PLAGUE SITUATION at San Francisco grows more serious and constitutes a grave menace to the whole country. Dr. Kinyoun, of the Marine Hospital Service, one of the ablest, if not the ablest, bacteriologist in the service, is still on the ground; but we have no report coming from him later than that published in the Marine Hospital Service Public Health Reports (weekly) of November

23 (ult.). According to this,—which is official,—from March 7 to November 4 (ult.) there had occurred twenty-three cases of the plague ,all of which but two were fatal. It is next to impossible to get reliable information of the situation from any other source than these Marine Hospital Service reports, and in them the details are very meagre. The State Health Officer of Texas is very active in measures for the protection of this State, and has placed on duty competent inspectors at all points at which the disease could be brought into the State. He has a representative at San Francisco, who will be vigilant, and will keep him thoroughly informed as to the progress of the disease. The railroads are lending their hearty co-operation to the State Health Officer in the matter, and every precaution is being taken to exclude the disease without having to lock the wheels and put the veto on trade and travel altogether.

In this connection we may mention that Dr. Arthur S. Wolff, the oldest officer in the State public health service, who has been State Quarantine Officer at Brownsville twenty-six years, arrived in Austin December 2 on a summons from Dr. Blunt, the State Health Officer, who wished him to accept service in the plague war at some point, and it is contemplated to assign him to duty at San Francisco a little later, or at El Paso, Texas,—the officer from the latter place being now at San Francisco, and it is understood wishes to return to his post. It is of the highest importance that the State of Texas should have on duty at San Francisco an able, honest, experienced and fearless officer, and Dr. Wolff will fill all the requirements.

---

SMALLPOX has made its appearance amongst the inmates of the North Texas Insane Asylum at Terrell, Texas. The disease is of a mild form, and we learn that it is well under control at this writing.

The quarantine against smallpox on the Rio Grande seems to be a useless expenditure of money, as it does not, and cannot, prevent the introduction of the disease from Mexico into Texas. In the very nature of things, it is impossible. For several hundred miles along the whole length of the Texas frontier the river is fordable at almost any point at certain seasons of the year,—as at present, for instance; and the Mexican laboring people, who do not mind smallpox, but who, rather, expose themselves and their children to it, purposely, to "have it and be over with it," as they say, cross on foot, or on burros, or in wagons, at almost any point, thus evading the guards who are on duty *only* at the railroad crossings. It would require an army larger than that of the United States (on a "peace footing") to prevent it,—to guard the entire length of the border. The situation is like a fence which is down, except at about five places: it "isn't worth while." Then why pay five or ten officers $1800 a year each, all the year round, to mind these "gaps"? The only rational way to deal with the situation is for Texas to enact a compulsory vaccination law, and see that it is enforced. By the bye, if we are not mistaken the United States immigration laws prohibit the entrance into the United States of any person who has not been vaccinated. Whose duty is it to see that this law is not evaded on the Mexican border? The Marine Hospital Service should look into the matter.

---

Yellow fever is prevailing in a mild form in Mississippi—at Brookhaven and at Natchez. There is no danger of an epidemic at this season, and Texas has not put on quarantine.

Speaking of this matter, the *Mississippi Medical Record* (Vicksburg), in a spirited editorial (which we "lift" bodily), says:

That yellow fever has again been introduced in Mississippi there can be no doubt. On the 9th of November Dr. Brown, of the Natchez Hospital, in the presence of Dr. H. A. Gant, president of the Mississippi State Board of Health; Dr. Wertenbaker, of the Marine Hospital Service, and a number of the local physicians, conducted a post-mortem examination that showed conclusively the character of the trouble, and the diagnosis of yellow fever was unanimously concurred in by all present.

At a subsequent meeting held by these gentlemen in the Natchez Hotel, it was practically agreed, in the light of subsequent events, that the introduction of the disease in Natchez occurred about the middle of September, and up to the time of this meeting there had been at least fourteen cases and possibly many more; positively seven deaths and possibly three others. There was practically no malignancy until a cool spell of some ten days' duration. It was then that some of the local physicians suspected the trouble. After this it was not many days before the true character of the disease, by unmistakable symptoms, asserted itself.

Without an absolute knowledge of the origin of the fever it is unfair to point with suspicion to any town whence it might have come.

There is one thing certain—made plain by the events of the last few years: Human life is at a discount when staked against a dollar. Each year brings us nearer to a time when the South will be visited by an epidemic that will send hundreds and thousands of her citizens to untimely graves. Appropriations can not prevent it; the trouble is behind all this. As we have said before, commerce is stronger before the public than sanitation. Everywhere, sanitarians,—national, State and local, yield to the demands of trade, and trade has no regard for human life when "pitted" against the "almighty dollar."

Yellow fever is not indiginous to the Southern States, and but for commercial relations with countries south of us, with a maritime quarantine practically annihilated by the demands of commerce, yellow fever would be unknown in any part of the United States.

Under the present system of maritime quarantine our danger is as great from the North as from the South. On account of quarantine regulations a large portion of the freight and passenger traffic has been deflected from Southern to Northern ports. This pressure on Southern Commerce has forced on Southern maritime quarantine so short a detention of vessels from infected ports that it is inconsistent with good sanitation, and each year is a menace to the health of the South. Again, quarantine detention of vessels from infected ports should be just as long for Northern as for Southern ports if maritime quarantine is intended for the protection of the whole country. If a vessel is disinfected in the port of New York and the crew and passengers at once released they can immediately come South by rail, develop fever and thus endanger the country. If a person is exposed to the infection of yellow fever on board a vessel, it matters not in what climate, he is not safe until he has passed the incubative period of the disease, dating from the time of disinfection; and since the incubative period is from three to five days he has ample time, after disinfection in New York to come South and develop fever in New Orleans before the period of incubation has passed.

It is pretty well known by the health authorities that Natchez is not the only town in this State this season that has been infected with yellow fever, and that has had deaths from the disease; but what can they do? What does a death from a preventable disease amount to, anyway? What does a hundred or even a thousand amount to in comparison to the disinfection, or a two days' longer detention of an ocean steamer, or even the sale of a bunch of bananas? The man who throws the weight of his influence against correct sanitary principles, regardless of the interest he is thereby serving, is responsible to a degree commensurate with his influence for every death that a violation of these principles may cause.

